# Low Resolution Solution Structure of HAMLET and the Importance of Its Alpha-Domains in Tumoricidal Activity

**DOI:** 10.1371/journal.pone.0053051

**Published:** 2012-12-27

**Authors:** James Ho CS, Anna Rydstrom, Malathy Sony Subramanian Manimekalai, Catharina Svanborg, Gerhard Grüber

**Affiliations:** 1 Department of Microbiology, Immunology and Glycobiology (MIG), Institute of Laboratory Medicine, Lund University, Lund, Sweden; 2 School of Biological Sciences, Nanyang Technological University, Singapore, Republic of Singapore; University of Oulu, Finland

## Abstract

HAMLET (Human Alpha-lactalbumin Made LEthal to Tumor cells) is the first member in a new family of protein-lipid complexes with broad tumoricidal activity. Elucidating the molecular structure and the domains crucial for HAMLET formation is fundamental for understanding its tumoricidal function. Here we present the low-resolution solution structure of the complex of oleic acid bound HAMLET, derived from small angle X-ray scattering data. HAMLET shows a two-domain conformation with a large globular domain and an extended part of about 2.22 nm in length and 1.29 nm width. The structure has been superimposed into the related crystallographic structure of human α-lactalbumin, revealing that the major part of α-lactalbumin accommodates well in the shape of HAMLET. However, the C-terminal residues from L105 to L123 of the crystal structure of the human α-lactalbumin do not fit well into the HAMLET structure, resulting in an extended conformation in HAMLET, proposed to be required to form the tumoricidal active HAMLET complex with oleic acid. Consistent with this low resolution structure, we identified biologically active peptide epitopes in the globular as well as the extended domains of HAMLET. Peptides covering the alpha1 and alpha2 domains of the protein triggered rapid ion fluxes in the presence of sodium oleate and were internalized by tumor cells, causing rapid and sustained changes in cell morphology. The alpha peptide-oleate bound forms also triggered tumor cell death with comparable efficiency as HAMLET. In addition, shorter peptides corresponding to those domains are biologically active. These findings provide novel insights into the structural prerequisites for the dramatic effects of HAMLET on tumor cells.

## Introduction

HAMLET (**H**uman **A**lpha-lactalbumin **M**ade **LE**thal to **T**umor cells) kills a wide range of tumor cells *in vitro*, including carcinoma and lymphoma cells from different species and has shown therapeutic efficacy against glioblastomas, papillomas and bladder cancer *in vivo* [1]–[3]. Healthy, differentiated cells are much less sensitive to HAMLET and toxic effects on healthy tissues have not been observed in tumor models. While the molecular basis of this difference in sensitivity is not entirely understood, the sensitivity to HAMLET has been shown to reflect *c-myc* oncogene expression and the glycolytic state of tumor cells [4]. Tumor cells have also been shown to internalize HAMLET more efficiently than healthy differentiated cells and once the intracellular HAMLET reaches different molecular targets in mitochondria, proteasomes and nuclei, several critical cellular functions are perturbed, leading to cell death [5]–[9].

HAMLET is formed from α-lactalbumin after partial unfolding of the protein and binding of oleic acid, with a stoichiometry of 4–8 fatty acid residues per protein molecule [10]. The tumoricidal activity of HAMLET was discovered in the casein fraction obtained after low pH precipitation of human milk. Since then, the HAMLET complex is formed by first purifying α-lactalbumin from human milk, unfolding by release of Ca^2+^ with EDTA, addition to an oleic acid-conditioned ion exchange matrix and elution with a step-wise NaCl gradient [6], [11]. Other similar protein-lipid complexes have also been reported, including BAMLET, ELOA and β-lactoglobulin-oleate complex [12]–[14]. It has been proposed that in the HAMLET complex, α-lactalbumin retains a molten globule like tertiary conformation also in the presence of calcium and at physiologic solvent conditions [11]. While α-lactalbumin acts as the glucose specifier for β-1,4-galactosyltransferase [15], [16], the native protein does not form tumoricidal complexes with oleic acid, demonstrating that partial unfolding of the protein alters its activity. Based on these findings, we have proposed that proteins may respond to different environments by changing their fold and binding partners and that this process allows a single polypeptide chain to exert vastly different biologic functions in different tissue compartments [11]. Furthermore, HAMLET exemplifies how protein unfolding may be beneficial and not just a cause of toxic amyloid formation.

The structural basis for HAMLET's tumoricidal activity is not fully understood. Studies of human α-lactalbumin lacking all disulphide bridges (rHLA^all-ALA^-OA) [10] show that protein unfolding alone is not sufficient to kill tumor cells and oleic acid alone is not toxic in the molecular range, where the complex kills tumor cells [10], [17]. This suggests that partial unfolding alters the protein structure to facilitate fatty acid binding and molecular interactions that initiate cell death. Since native α-lactalbumin lacks these activities, we hypothesized that the partially unfolded and fatty acid bound form of α-lactalbumin might expose novel epitopes for tumor cell interaction. In this study, we have solved the HAMLET solution structure at low resolution and have mapped exposed epitopes for contributions to the tumoricidal activity. We have also constructed a functional peptide map in the presence of sodium oleate, using cellular uptake, holography, ion channel activation and cell death. These results show that α-helical domains of HAMLET interact with tumor cells in the presence of sodium oleate and that they trigger many of the cellular responses seen in HAMLET treated cells.

## Materials and Methods

### Production of HAMLET

α-Lactalbumin was purified from human breast milk by ammonium sulphate precipitate and hydrophobic interaction chromatography. The purified α-lactalbumin was partially unfolded by EDTA treatment subjected to DEAE Trisacryl M matrix preconditioned with oleic acid and converted to HAMLET by removal of calcium and binding to oleic acid as previously described [11]. Human milk was obtained from individual donors, after signed informed consent. Each donor was aware that the samples may be used in scientific research. The samples were de-identified and steps were taken to protect the participants' identities. The procedure was approved by the human ethics committee of the Medical Faculty, Lund University, Lund, Sweden.

### X-ray scattering experiments and data analysis of HAMLET

Small angle X-ray scattering (SAXS) data for HAMLET were collected at the German Electron Synchrotron (DESY) of the EMBL Hamburg using the X33 SAXS camera [18], [19] located on a bending magnet (sector D) on the storage ring DORIS III. Pilatus 1 M pixel detector (67×420 mm^2^) that operates in single photon counting mode was used. A sample - detector distance of 2.4 m which covers the range of momentum transfer 0.1<s<4.5 nm^−1^ (s = 4π sin(q)/λ, where q is the scattering angle and λ = 0.15 nm is the X-ray wavelength) was utilized. The calibration of the s-axis was done by the scattering pattern of Silver-behenate salt (d-spacing 5.84 nm). For each sample measurement the scattering from the buffer was measured before and after. The average scattering for the before and after buffer measured was calculated and it is used for background subtraction for each sample. In order to assess and remove any concentration-dependant inter-particle effects for HAMLET, the protein concentrations 2.5 and 6.5 mg/ml in phosphate-buffered saline were used for the measurement. Automated sample-changing robot was used for injecting the protein as well as the buffer samples at the SAXS station X33 [20]. All the data were processed using the program package PRIMUS [21] automatically. Guinier approximation [22] was used to evaluate the forward scattering *I*(0) and the radius of gyration *R_g_* which assumes that for a spherical particles at very small angles (*s<1.3/R_g_*) the intensity is represented by *I(s) = I*(0) *exp(-(sR_g_)^2^/3)*. The indirect transform package GNOM [23] was also used for computing these parameters for the entire scattering patterns as well as the distance distribution function *ρ(r)*.

By comparing the forward scattering from the reference solution of bovine serum albumin (BSA), the molecular mass of HAMLET was calculated as described in [18]. *Ab initio* low-resolution models for HAMLET were built by the program GASBOR [23]. In order to compare the solution structure of HAMLET with the atomic structure of human α-lactalbumin, the high resolution model (PDB entry 1B9O [24]) has been aligned using SUBCOMB [23]. This program aligns all possible pairs of models and arranges the smallest average discrepancy among the models. The data collection and scattering-derived parameters are given in Table 1.

**Table 1:** Data-collection and scattering-derived parameters.

Data-collection parameters	
Wavelength (nm)	0.15
s range (nm^−1^)	0.1–4.5
Exposure time (min)	2
Concentration (mg ml^−1^)	2.5 and 6.5
Temperature (°C)	15
Structural parameters	
I(0) (cm^−1^) [from P(r)]	13.46±0.04
R_g_ (nm) [from P(r)]	1.78±0.05
I(0) (cm^−1^) [from Guinier]	14.65±0.01
R_g_ (nm) [from Guinier]	2.08±0.02
D_max_ (nm)	5.69±0.1
Porod volume estimate (Å^3^)	20 876
Dry volume calculated from sequence (Å^3^)	17 100
Molecular-mass determination	
Molecular mass M_r_ [from I(0)]	15 000±2 000
Software employed	
Data processing	PRIMUS
*Ab initio* analysis	GASBOR
Validation and averaging	DAMAVER
Computation of model intensities	CRYSOL
Three-dimensional graphic representation	PyMOL

### Peptide synthesis

The designed peptides were synthesized by Mimotopes Pty Ltd, Melbourne, Australia. The peptides were synthesized using the mild Fmoc chemistry method. For biotinyalated peptides, an aminohexanoic acid (Ahx) spacer was added to ensure adequate separation between the biotin and the peptide moiety. The primary sequence follows the residue numbering in human α-lactalbumin. The sequences for the three larger peptides are as such: alpha1: KQFTKAELSQLLKDIDGYGGIALPELIATMFHTSGYDTQGWG; beta: IVENNESTEYGLFQISNKLWAKSSQVPQSRNIADISADKF alpha2: GWGLDDDITDDIMAAKKILDIKGIDYWLAHKALATEKLEQWLAEKL; peptide1: KQFTKAELSQLLKDI; peptide2: LLKDIDGYGGIALPE; peptide3: IALPELIATMFHTSG; peptide4: FHTSGYDTQAIVENN; peptide5: IVENNESTEYGLFQI; peptide6: GLFQISNKLWAKSSQ; peptide7: AKSSQVPQSRNIADI; peptide8: NIADISADKFLDDDI; peptide9: LDDDITDDIMAAKKI; peptide10: AAKKILDIKGIDYWL; peptide11: IDYWLAHKALATEKL; peptide12: ATEKLEQWLAEKL. GWG is a linker added to the construct to facilitate concentration determination for alpha1. This tripeptide linker has been used previously [25] in a minimized α domain molecule of α-lactalbumin (known as MinLeu), whereby all hydrophobic residues were substituted with leucine, and it had been shown to retain the secondary structural features of the native α-lactalbumin, thus it has minimal effect on the structure. For consistency, the linker is also added to alpha2. The 15-amino-acid peptides were numbered from the N-terminal. Purified peptides were assessed by analytical reverse phase HPLC (RP-HPLC) and checked for the correct identity by electrospray mass spectrometry (ESMS). The peptides were stored at −20°C upon delivery and stored lyophilized before use.

### Cellular assays

A549 human lung carcinoma cell line and the Jurkat human acute T-cell leukemia cell line (obtained from ATCC, Manassas, VA, USA) were cultured in RPMI-1640 with non-essential amino acids (1∶100), 1 mM sodium pyruvate (all from PAA, Pasching, Austria), 50 mg/ml Gentamicin (Gibco, Paisley, UK) and 5% fetal calf serum (FCS) at 37°C, 5% CO_2_. Cells (5*10^5^ cells/ml for A549 cells, 1*10^6^ cells/ml for Jurkat cells) in suspension were incubated with either HAMLET or peptide-oleate mixtures in serum-free RPMI-1640 at 37°C, 5% CO_2_. 5% FCS was added after 1 hour. Cell death was quantified by ATP level measurement (ATPlite Kit, PerkinElmer, Waltham, MA, Infinite F200, Tecan Group Ltd., Männedorf, Switzerland) and by using PrestoBlueTM Cell Viability Reagent (Invitrogen, Carlsbad, CA).

### Confocal microscopy

For cellular uptake assays, A549 lung carcinoma cells were grown overnight on 8-well chamber slides (Nalge Nunc, Rochester, NY). Cells were incubated 1 hour with 35 µM biotinylated peptide with or without 175 µM sodium oleate (Sigma) in serum free RPMI media, followed by fixation (4% paraformaldehyde) and permeabilization (0.25% Triton X-100, 5% FCS in phosphate-buffered saline). Peptides were visualized by incubation with streptavidin-Alexa568 (Molecular Probes) and Wheat Germ Agglutinin (WGA) (Molecular Probes) was used to visualize plasma and nuclear membranes. Images were captured on a LSM510 META confocal microscope (Carl Zeiss, Jena, Germany) with pinhole settings corresponding to one airy unit. Quantification of fluorescence intensities was performed with the ImageJ software.

### Phase Holographic Imaging

The HoloMonitor™ M3 digital holographic microscope (Phase Holographic Imaging AB, Lund, Sweden) records 3D information of cells using interfering wave fronts induced by the exposure to a 0.8 mW HeNe laser (633 nm), resembling a Mach-Zender interferometer where the cells, placed in one of the interfering wave fronts, induce a phase difference between the two beams [26]–[28]. The interference pattern (hologram) is recorded on a digital sensor and is used to reconstruct the amplitude and phase of the object as described [29], [30]. 40 000 A549 cells plated on μ-Slide I coated with ibiTreat (ibidi, Martinsried, Germany) were treated with HAMLET or the peptide-oleate mixtures and were incubated at 37°C, 5% CO_2_. The images were captured with an imaging time of 2.4 msec, every 15-minute interval.

### Ion flux measurement

To quantify the ion fluxes, fluorometry was measured in a plate reader (TECAN infinite F200, Tecan Group, Switzerland). For calcium fluxes measurement, the Fluo-4 NW calcium assay kit (Invitrogen) was used. For K^+^ fluxes, the FluxOR™ potassium ion channel assay (Invitrogen) was used according to the manufacturer's instructions but component E was excluded. Briefly, cells were incubated with FluxOR™, which is a Tl^+^ indicator. An increase in fluorescence signal corresponds to an influx of Tl^+^, indicating opening of potassium channels, which was measured at 535 nm after excitation at 485 nm in the TECAN infinite plate reader. Given the driving force for K^+^ flux across the plasma membrane this corresponds to K^+^ efflux. Calcium and potassium fluxes was measured in 20 000 A549 cells plated in a 96-well plate. Sodium was measured in 100 000 Jurkat cells using the CoroNa Green Sodium Indicator (Invitrogen).

## Results

### Low resolution solution structure of HAMLET

**Figure 1:**
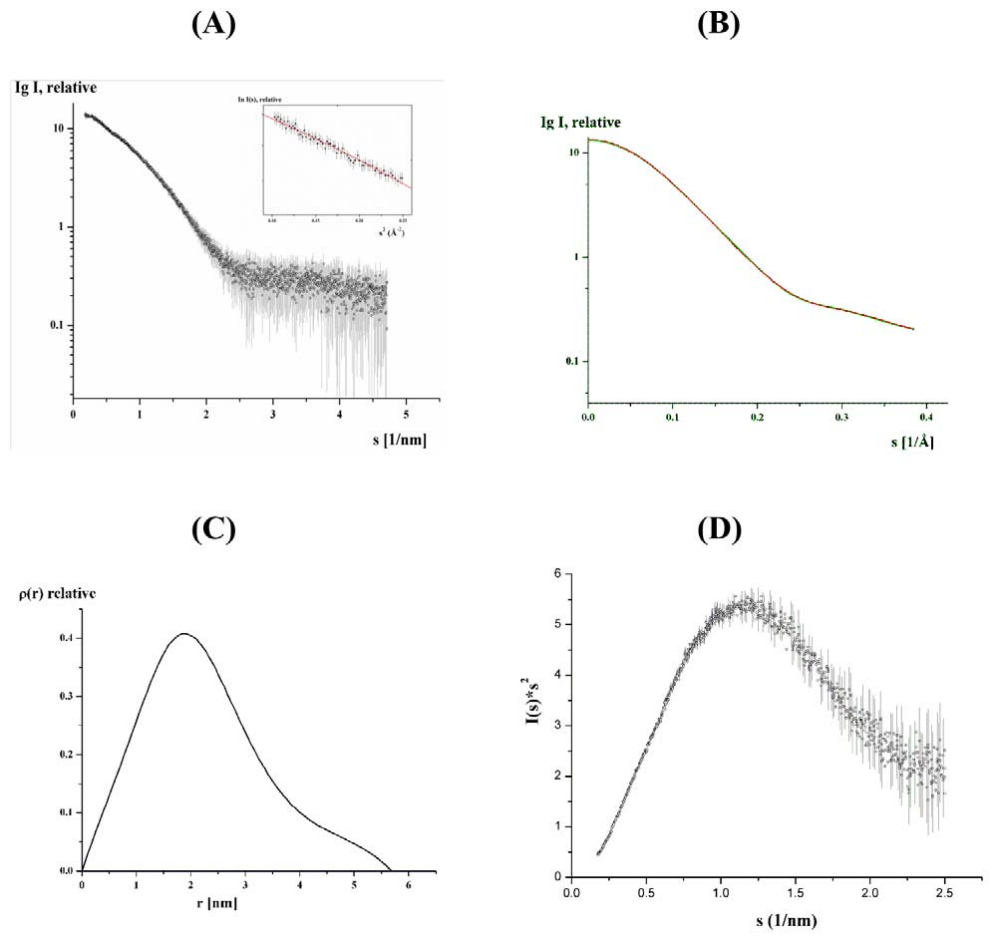
SAXS structure analysis. (A) Experimental scattering curves (o) with error bars in gray color and (*insert*) the Guinier plot with the linear fit (red line) are shown. (B) The fitting curves (—; *green*: fit for the experimental data, *red*: fit for the calculated *ab initio* model) of HAMLET derived from SAXS data. (C) Distance distribution functions of HAMLET. (D) The Kratky plot indicates that the protein is globular and folded. The increase at higher angles might reflect that the protein is slightly flexible.

The high purity of HAMLET enabled us to perform small-angle X-ray scattering experiments, to verify the proper three dimensional folding and to determine the first low resolution structure of this protein. SAXS patterns of HAMLET were recorded as described in Materials and Methods to yield the final composite scattering curve shown in figure 1A, which indicates a monodispersed protein in solution. Inspection of the low angle of the Guinier plots reveals a good data quality and no protein aggregation (*inset* of Fig. 1A). HAMLET has a radius of gyration (*R_g_*) of 1.78±0.05 nm and a maximum dimension *D_max_* of 5.69±0.1 nm (Fig. 1C). Comparison of the forward scattering of HAMLET with the values obtained from a reference solution of bovine serum albumin, (BSA; 66.4±2 kDa) yields a molecular mass of 15±2 kDa, in agreement with the mass of α-lactalbumin (24), indicating that HAMLET is monomeric at the concentrations used. Qualitative analysis of the distance distribution function suggests that HAMLET consists of a major portion, yielding a principal maximum in the *p(r)* around 1.9 nm (Fig. 1C), whereas the separated protuberance domain giving rise to a shoulder from 3.9 nm to 5.7 nm. The Kratky plot reveals that the protein is globular and folded (Fig. 1D). The increase in higher angles in the plot might indicate that the protein is slightly flexible.

In a complementary approach the native molecular mass of HAMLET was determined via size exclusion chromatography. A Superdex 75 gel filtration column was calibrated by determining the *K_av_* values for a set of standard proteins of known MM (Fig. S1). A calibration curve based on these *K_av_* values is shown in figure S1B. Comparison of the *K_av_* for HAMLET *versus* the standard proteins suggests the native molecular mass (MM) of approximately 16±2 kDa.

**Figure 2:**
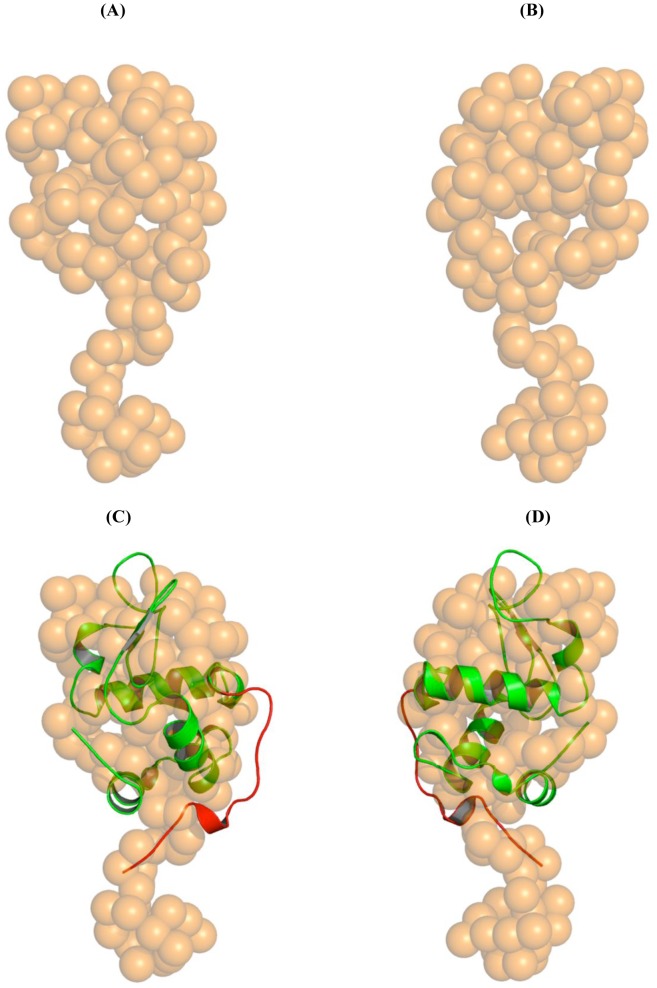
SAXS structure of HAMLET and native human α-lactalbumin crystal structure. (*A*) Low resolution structures of HAMLET in brown spheres and (*B*) its 180° view. (*C–D*) Superposition of HAMLET with human α-lactalbumin (PDB id: 1B9O [21]). The C-terminal residues from L105 to L123 of the crystal structure of the human α-lactalbumin, which form a flexible loop in the crystal structure of human α-lactalbumin, are colored red. We suggest that this region L105 to L123 takes up an extended conformation in HAMLET by forming a tail.

The solution structure of HAMLET was restored *ab initio* from the scattering patterns, shown in figure 1A. The obtained shape for the protein yields a good fit to the experimental data in the entire scattering range. The corresponding fit, shown in figure 1B, has a discrepancies of χ^2^ = 1.36. The protein appears as a two domain molecule with a large globular domain and an extended part of about 2.22 nm in length and 1.29 nm width (Fig. 2A–B). The major domain has dimensions of about 3.16×2.57 nm.

Superimposing the crystallographic structure of human α-lactalbumin (PDB id: 1B9O) into the low resolution solution structure of HAMLET shows that the major part of α-lactalbumin accommodates well in the shape of HAMLET with an r.m.s. deviation of 0.11 nm (Fig. 2C–D). Superposing other orientations of the α-lactalbumin, such us the N-terminus near the extended part, yielded larger deviations (r.m.s. deviation being in the range 0.15–0.25 nm). Finally, only the orientation with the C-terminus facing the extended domain gave least deviation, which demonstrates that the extended part of the shape belongs to the C-terminal region. The experimental curve fits with the theoretical scattering curve of crystal structure (PDB id: 1B9O) with a χ^2^ of 1.531 as calculated by CRYSOL program. Figure 2C reveals that the C-terminal residues from L105 to L123 of the crystal structure of the human α-lactalbumin does not fit well into the low resolution model. This region forms a flexible loop in the crystal structure of human α-lactalbumin and is held in place by interactions with the symmetry related molecules due to crystal packing. We propose that this region L105 to L123 takes up an extended conformation in HAMLET by forming a tail. This structural alteration may be a result of the predicted partial unfolding and fatty acid binding, required to form HAMLET [10].

### Effects of HAMLET peptides on tumor cells

**Figure 3:**
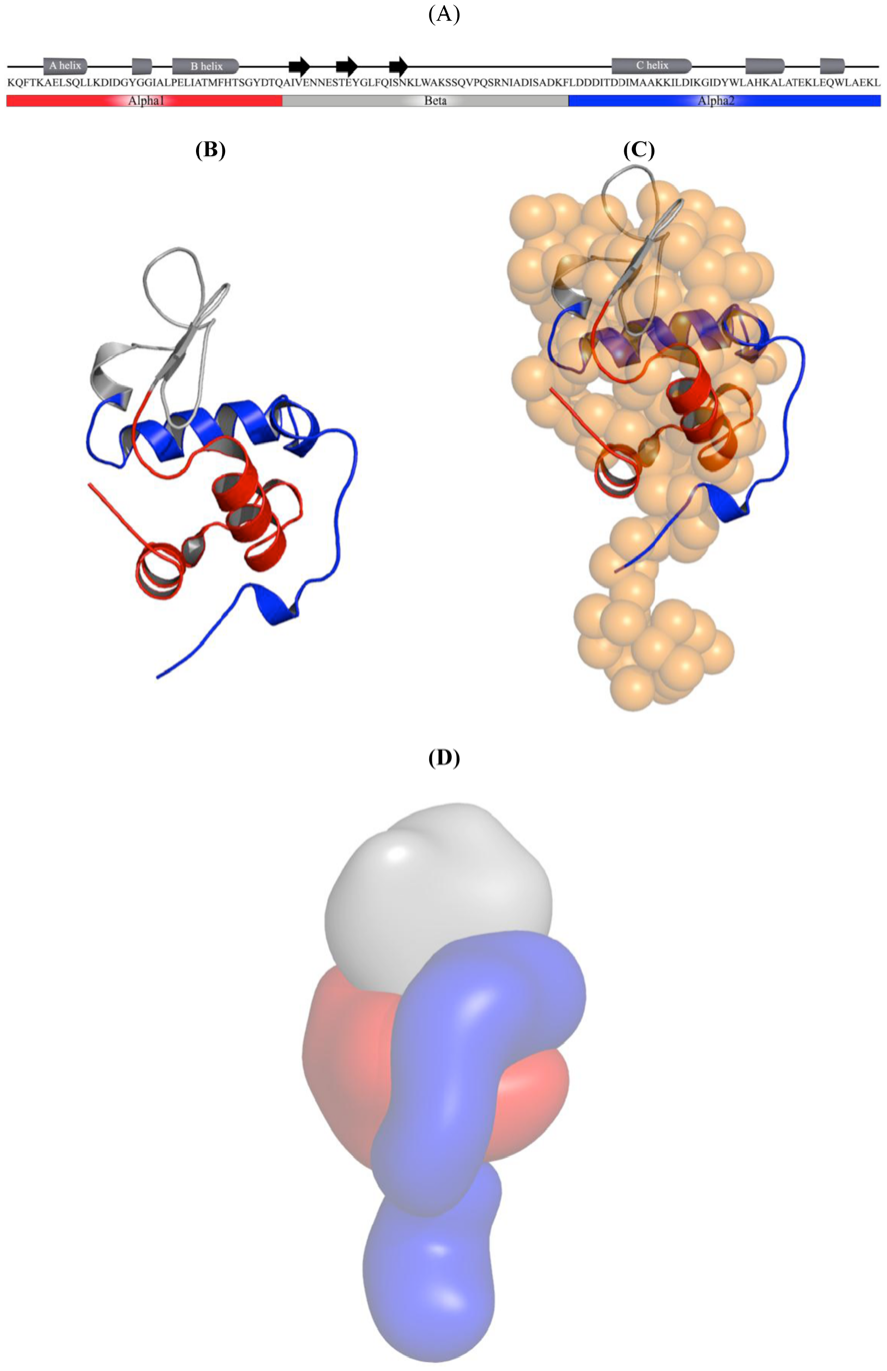
Peptide sequences. (A) Amino acid sequence and secondary structure of human α-lactalbumin as well as the peptide domains alpha1, alpha2 and beta. (B) The three peptide domains in the crystallographic structure of human α-lactalbumin (PDB id: 1B9O [21]) (C), which is superimposed into the solution shape of HAMLET (C). (D) Surface representation showing the low resolution structure of the human α-lactalbumin with the three domains; alpha 1 in red, alpha 2 in blue and beta in gray color. The tail region of the SAXS shape, which is modeled on to the alpha2 domain low resolution structure, is also shown in blue.

Recent Far-UV CD spectroscopy studies have shown that the secondary structural features in HAMLET are similar to those in α-lactalbumin [10], which include an alpha1 and alpha2 domain, consisting of the amino acid residues K1-Q39 and L81-L123, respectively, as well as the beta domain, formed by the residues A40-F80 (Fig. 3A–B). The first low resolution solution structure of HAMLET shows that the major domain of HAMLET is composed by the alpha1 and beta domain. As described above the alpha2 domain forms a part of the globular domain (residues L81 to W104) as well as the C-terminal tail with the amino acids L105 to L123. In order to identify epitopes involved in the biological effects of the HAMLET, the peptides of the three domains alpha1, alpha2 and beta were synthesized (Fig. 3A) with substitutions of alanine to cysteine residues. The design of peptides took our previous work into consideration, which demonstrated that recombinant human α-lactalbumin mutant (rHLA^All-ala^), when deprived of all disulphide bridges by cysteine to alanine substitution, retains the ability to form a complex (rHLA^All-ala^-OA) with full tumoricidal activity, similar to HAMLET [10].

**Figure 4:**
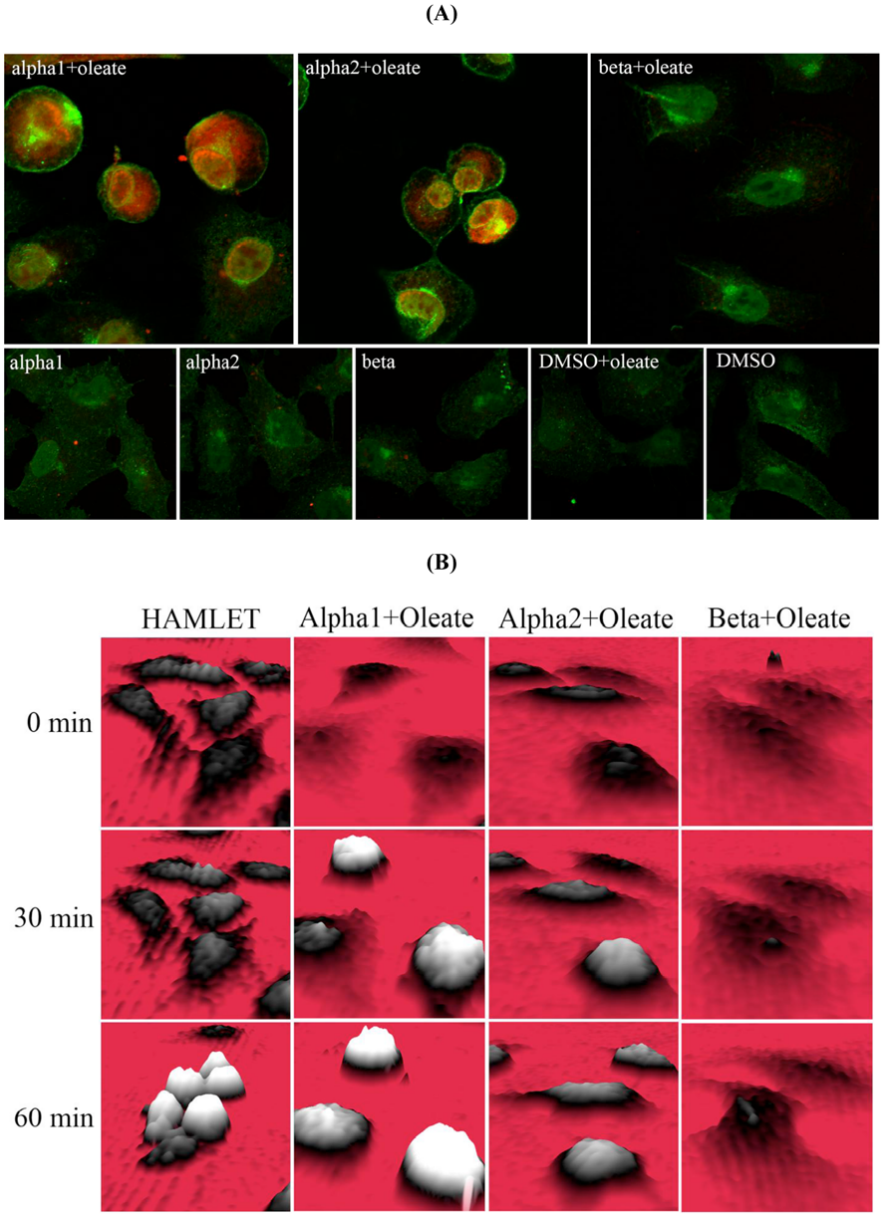
Internalization of peptides into tumor cells and changes in tumor cell morphology. (A) Internalization of peptides. A549 lung carcinoma cells cultured on glass slides, were incubated with peptide-oleate mixtures for 1 hour, fixed and stained with AlexaFluor568-streptavidin, counterstained with WGA and examined by confocal microscopy. Alpha1 and alpha2 peptides, mixed with oleate, were internalized as shown by the red fluorescence. The beta peptide was not internalized. Scale bar 20 µm. (B) Morphological changes in A549 lung carcinoma cells treated with HAMLET, alpha1 peptide+oleate, alpha2 peptide+oleate and beta peptide+oleate recorded by holography imaging. Cells treated with HAMLET started to round up after 30 minutes and after 60 minutes, many cells had detached. Alpha1 peptide+oleate mixture triggers similar morphological changes as that by HAMLET. Alpha2 peptide+oleate mixture triggers similar morphological changes. Beta peptide+oleate mixture did not change cell morphology.

To analyze the cellular uptake of the alpha1, alpha2 and beta peptides, lung carcinoma cells were exposed to biotinylated peptides in the presence or absence of sodium oleate, and counterstained with AlexaFluor568-conjugated Steptavidin for confocal microscopy visualization (Fig. 4A). The peptide-oleate complex was prepared by mixing 35 µM of peptide with 175 µM of sodium oleate prior to the incubation with tumor cells. A decrease in the turbidity of the sodium oleate solution upon mixing with the peptide solution indicates the complexation of sodium oleate with the peptide, as previously reported in other systems [31], [32].

In the oleate-bound form, the alpha1 peptide was internalized showing a uniform cytoplasmic staining pattern and nuclear accumulation. In addition, the peptide accumulated in the nuclear periphery, adjacent to the inner leaflet of the nuclear membrane, as shown by counterstaining with WGA, which stains cytoplasmic and nuclear membranes [33]–[35]. The fluorescence intensity reflecting peptide internalization was increased 6-fold compared to the Streptavidin/dimethyl sulfoxide (DMSO) control (P<0.001) (Fig. S3). In comparison, no significant internalization of the peptide was observed in the absence of sodium oleate. The alpha1 peptide-oleate complex was also shown to influence cell morphology. Transmission light differential interference contrast (DIC) images (Fig. S2) revealed that the cells rounded up, while cells exposed to the peptides alone maintained the extended morphology of the control cells.

By holographic imaging, which constructs a 3D image from modified interference patterns, the temporal changes in cellular shape and density were followed in real time, without fixation of the cells. Adherent cells, exposed to HAMLET showed an increase in cellular optical thickness and a reduction in adherent surface area after 30 minutes, followed by cell detachment at later time points. The alpha1 peptide-oleate complex caused a morphologic response with similar kinetics, but detachment was delayed compared to HAMLET (Fig. 4B).

The cellular interactions of the alpha2 peptide resembled those of the alpha1 peptide. The alpha2 peptide-oleate bound form was internalized with similar efficiency (6-fold increase in fluorescence intensity as compared to control, p<0.001), and with a similar staining pattern as the alpha1 peptide-oleate mixture (Fig. S3). Morphological changes were similar as observed by transmission DIC (Fig. S2). Holographic imaging of tumor cells treated with the alpha2 peptide-oleate mixture showed a rapid increase in cellular optical thickness and a reduction in adherent surface area after 30 minutes, followed by cell detachment at later time points, resembling the response to the alpha1-oleate complex (Fig. 4B).

In contrast, tumor cells treated with the beta peptide-oleate mixture showed no internalization of the beta peptide (Fig. 4A). In addition, both transmission DIC images (Fig. S2) and holographic images (Fig. 4B) showed that the cells remained morphologically intact and fully adherent.

### Peptide-oleate mixtures trigger ion fluxes in tumor cells

**Figure 5:**
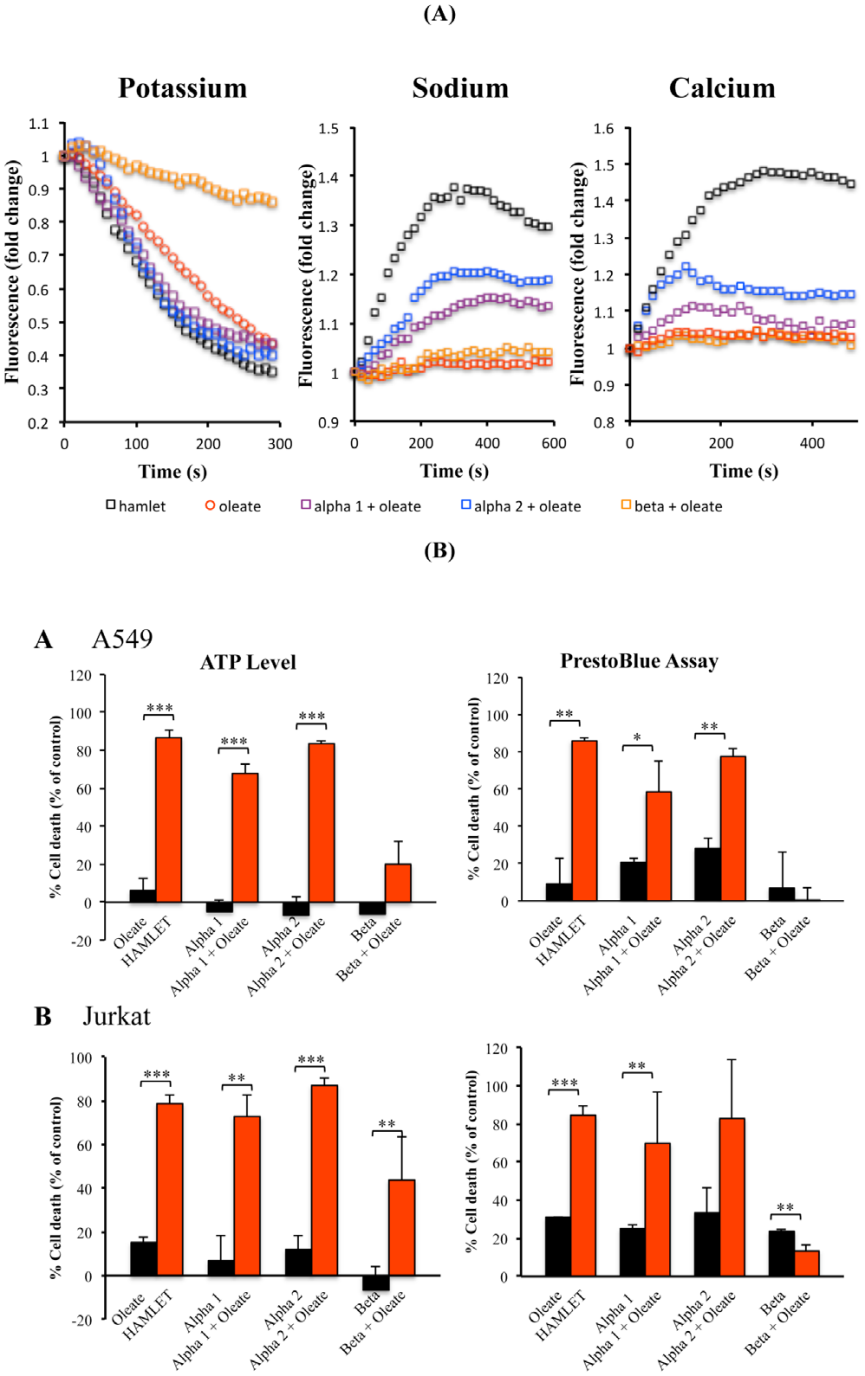
Ion fluxes and tumor cell death. (A) Peptides trigger ion fluxes in tumor cells. The free intracellular concentration of Na^+^, K^+^ and Ca^2+^ were measured by fluorescence spectrometry using CoroNa Green, FluxOR and Fluo-4, respectively. HAMLET, alpha1 peptide+oleate and alpha2 peptide+oleate mixtures trigger rapid fluxes of all three ions. Intracellular potassium ion concentrations were reduced due to ion efflux, while those of sodium and calcium were increased. Mean of at least two experiments. P values are explained in the text. (B) Peptide-oleate mixtures kill tumor cells. A549 lung carcinoma cells and Jurkat leukemia cells were incubated with HAMLET, oleate or peptide-oleate mixtures for 3 hours. Cell death was quantified as ATP levels and PrestoBlue, in % of control.

HAMLET triggers rapid and sustained K^+^ and Ca^2+^ fluxes in lung carcinoma cells and Na^+^ fluxes in Jurkat cells (unpublished). To identify HAMLET peptide epitopes responsible for ion channel activation, cells were preloaded with Na^+^, K^+^, or Ca^2+^ fluorophores and exposed to peptides or peptide-oleate mixtures. Changes in intracellular ion concentrations were characterized by fluorometry. The alpha1-oleate mixture triggered a brief Ca^2+^ flux and sustained Na^+^ fluxes (p<0.01), which were weaker than responses to HAMLET (p<0.05) (Fig. 5A). Both, the alpha1- as well as the alpha2-oleate complex stimulated K^+^ fluxes, which were similar to those triggered by HAMLET. The K^+^ efflux was monitored as the influx of thallium, which is used as a surrogate marker for K^+^.

In comparison, the alpha2 peptide-oleate mixture triggered a profile of ion fluxes, which resembled that of the alpha1 peptide-oleate mixture and HAMLET. However, the Ca^2+^ fluxes caused by the alpha2 peptide-oleate mixture were weaker than for HAMLET (p<0.01).

Without sodium oleate, the alpha peptides were inactive. Sodium oleate alone triggered significant K^+^ fluxes but Ca^2+^ and Na^+^ fluxes were not observed. The beta peptide-oleate mixture suppressed the K^+^ flux triggered by sodium oleate alone (p<0.003) but did not modify Na^+^ or Ca^2+^ fluxes.

### Peptide-oleate mixtures trigger tumor cell death

To examine, if the peptide and sodium oleate mixtures have tumoricidal activity, lung carcinoma or leukemia cells were exposed to each peptide with or without sodium oleate and cell death was quantified as a reduction in ATP levels or decrease in PrestoBlue fluorescence. HAMLET was used as a positive control, reducing cell viability by 90% after 3 hours. The alpha1 peptide triggered cell death, when mixed with sodium oleate (Fig. 5B), reducing cell viability by about 60% after 3 hours in lung carcinoma (p<0.001) and leukemia cells (p<0.05). In analogy, the alpha2 peptide triggered cell death when mixed with sodium oleate. Viability was reduced by about 80% after 3 hours (p<0.001). This tumoricidal effect was lower than for HAMLET (p<0.05 compared to alpha1 peptide-oleate mixture and p<0.06 for alpha2 peptide-oleate mixture). The beta peptide-oleate mixture showed no significant effects on the viability of lung carcinoma cells but marginally reduced the ATP levels in Jurkat cells (p<0.05). In the absence of peptide, sodium oleate alone did not kill the tumor cells (p<0.001 compared to HAMLET, p<0.002 for alpha1-oleate, p<0.001 compared to alpha2-oleate).

Taken together, the results suggest that the alpha domain peptides reproduce essential aspects of the tumor cell response to HAMLET, when mixed with sodium oleate. The beta peptide, in contrast, failed to activate such responses and rather acted as an inhibitor, at least for K^+^ ion channel activation. The results also suggest that the tumoricidal effects of HAMLET are reproduced by alpha peptides mixed with sodium oleate, but not by the beta peptide or by sodium oleate alone.

### Extended peptide mapping

Following the identification of the alpha domain peptides as tumor cell death agonists, peptide mapping was extended using a library of biotinylated peptides from the N- to C-termini of α-lactalbumin, each 15 amino acids long with 5 amino acid overlaps (Fig. 3A). Peptides 1–4 corresponded to the alpha1-, peptides 5–8 to the beta- and peptides 9–12 to the alpha2 peptide.

**Figure 6:**
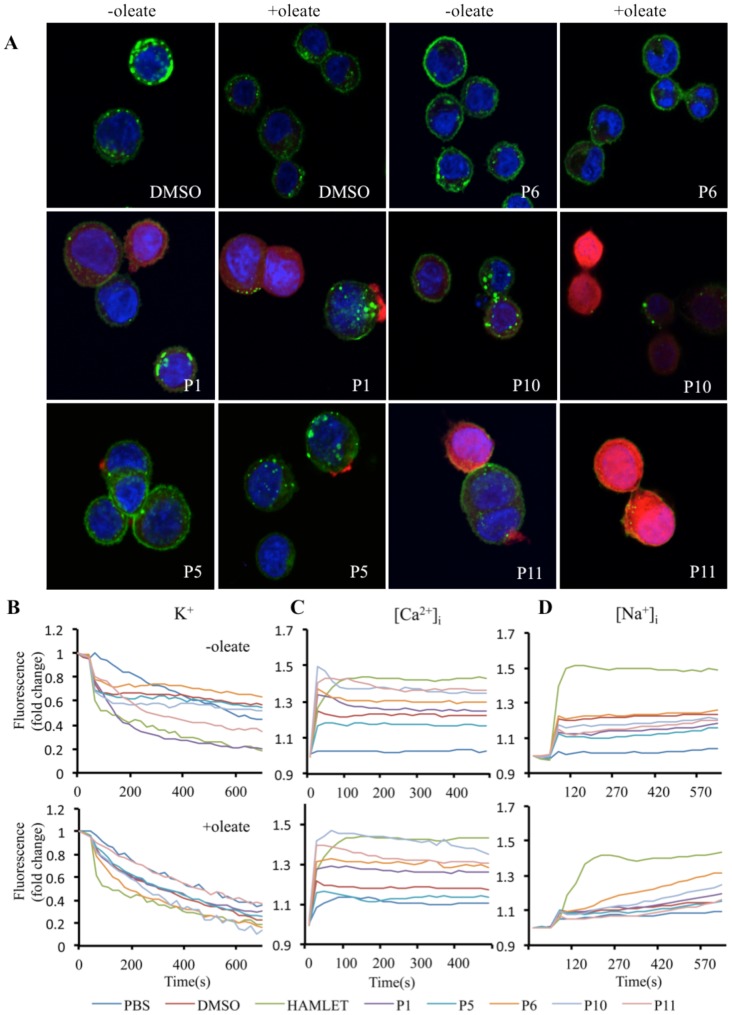
Internalization of small peptides and induction of ion fluxes. (A) Internalization of biotinylated peptides (red) by A549 lung carcinoma cells counterstained with WGA (green) and Hoechst (blue) and examined by confocal microscopy. Peptides 1,10 and 11 were internalized in the absence of oleate and peptides 10 and 11 also in the presence of oleate. (B) K^+^ and (C) Ca^2+^fluxes in tumor cells triggered by petides 1, 10 and 11 were measured by fluorescence spectrometry. Peptides 10 and 11 triggered Ca^2+^ fluxes, in the presence and absence of oleate and peptide 6 a weaker Ca^2+^ flux with oleate. (D) Insignificant Na^+^ fluxes.

To examine the cellular uptake, A549 lung carcinoma cells were treated with each peptide (105 uM, except for peptide 6, which was added at 12 µM due to its lower solubility) and probed with AlexaFluor568-conjugated Streptavidin for confocal microscopy visualization (Fig. 6A). Peptides 1, 10 and 11 showed significant internalization by the tumor cells and the strongest nuclear localization. This effect was enhanced in the presence of sodium oleate for peptides 10 and 11, but not for peptide 1, demonstrating that certain peptides have independent cell-penetrating properties. Remaining peptides were negative or internalized to a much lesser extent (Fig. S4).

The effects on ion fluxes were examined in fluorophore-preloaded cells (Fig. 6B–D). The two cell penetrating peptides 1 (p<0.01) and 11 (p<0.05) triggered K^+^ fluxes independent of sodium oleate. Peptide 11 also increased Ca^2+^ fluxes in the absence and presence of sodium oleate. Peptide10 induced a weaker Ca^2+^ flux in the absence and presence of sodium oleate, as did the peptide 6-oleate mixture. The baseline for K^+^ fluxes was increased by sodium oleate and all ion fluxes generally by DMSO, used to solubilize the biotinylated peptides. Weak Na^+^ fluxes were recorded for peptide 6 and peptide 10 when mixed with sodium oleate. K^+^ fluxes were comparable to those induced by HAMLET but Ca^2+^and Na^+^ fluxes were weaker. A summary of the effects of these peptides is included in table S1.

Cell viability was examined after a 3-hour exposure of lung carcinoma cells to the peptides alone or peptide-oleate mixtures. No tumoricidal activity was detected, suggesting that the combined effect of several domains of the protein is needed to trigger tumor cell death (Fig. S5).

## Discussion

HAMLET represents a new family of protein-lipid complexes with tumoricidal activity, whose structures have remained unsolved. It has also been debated to what extent the lipid and protein components of these complexes contribute to the cellular responses leading to death. The presented low-resolution solution structure of HAMLET shows that this protein has a two-domain feature in solution in contrast to its compact precursor protein α-lactalbumin. We suggest that the flexible loop at the C-terminal region of the protein enables the amino acids L105 to L123 to form an extended conformation in HAMLET and increase thereby the overall surface and accessibility for the lipid as well as the binding partner in the tumor cell (Fig. 3D).

Peptide mapping identified the alpha1 and alpha2 domains as tumor cell ligands, involved in internalization, ion channel activation and cell death. The SAXS structure of HAMLET predicted that both of these domains might become more accessible in HAMLET than in native α-lactalbumin. In the native state, the alpha2 domain folds back onto the alpha1 domain, thus creating the globular shape [21]. The extended conformation of the C-terminal residues from L105 to L123 in HAMLET may thus have the dual effect of exposing novel peptide epitopes in the alpha2 domain *per se* and of uncovering epitopes in the alpha1 domain, which normally are protected in the native protein structure. These findings extend and offer a structural context to the results of Tolin et al, using peptide digests and oleic acid [32]. As the fragments used did not exclusively cover the alpha or the beta domain, a generalization of the role of amino acid sequences led to the conclusion that oleic acid is the biologically active entity. Our results define the existence of at least two functional domains in alpha-lactalbumin that interact with sodium oleate and form tumoricidal complexes. The tumoricidal activity thus resides in both termini of the alpha domain but not the beta domain, demonstrating that specific peptides are involved. Further support for peptide specificity was obtained by Baumann et. al. [36], studying the differential interaction of alpha-lactalbumin fragments with phospholipid monolayers. In particular, Peptide A (residues 1–18) and Peptide C (75–100) showed positive interactions and annular HAMLET structures were observed on artificial lipid surfaces rather than the monomeric structure observed in solution. The molecular shapes of HAMLET, when in contact with host cells, remain to be defined.

The general cytotoxicity of unsaturated fatty acids is well recognized, including oleic acid [37]–[41]. High lipid concentrations are cytolytic and mitochondrial membrane depolarization by lipids may activate a caspase-independent cascade with inactivation of the Bcl-2-associated death promoter (BAD). HAMLET-like protein-lipid complexes produced by other methods, such as BAMLET (the HAMLET counterpart made from bovine alpha-lactalbumin) with much higher lipid-protein ratios [42], [43] may therefore have properties different from those of the defined HAMLET complex, which carries 5–8 oleic acid residues per protein molecule [10]. Importantly, oleic acid alone is not cytotoxic at the concentrations present in HAMLET and extensive comparisons between oleic acid and HAMLET treated cells, including genome-wide transcription analyses, have demonstrated that oleic acid *per se* is virtually inert, as opposed to HAMLET (unpublished). It is also important to note that other types of fatty acids are less efficient in forming cytotoxic complexes and less active than HAMLET [44], further suggesting that the protein and lipid are both required for the specific response to HAMLET and the increased effect on tumor cells.

Crystallographic and NMR structural comparisons between bovine apo- and holo-α-lactalbumin have revealed structural differences in the cleft between the two domains [45], [46]. The expansion in apo-α-lactalbumin of the Ca^2+^ binding loop tilts the 3_10_ helix towards the C helix and disrupts the hydrophobic box (aromatic cluster II) the interface between the two domains [24], which involves residues W26, F53, W60, Y103 and W104 [47], [48]. The current SAXS structure identifies additional structural alterations in HAMLET. The extended conformation of L105–L123 will inevitably alter the adjacent hydrophobic box and thereby expanding the solvent exposed hydrophobic surface compared to the native protein, as shown by increased 8-anilinonaphthalene-1-sulfonate (ANS) binding [10]. A secondary effect of this major structural alteration is in the exposure of the alpha domain, represented by the alpha1 peptide in the current study. Alterations in the alpha1 region potentially expose aromatic cluster I in α-lactalbumin [47], [49]. The biological activities shown by the alpha domain peptides together with sodium oleate suggest that both of these domains bind oleate and present the lipid to tumor cells in a unique way, not reproduced by either constituent alone. However, there was no evidence that exposure of the cleft region by structural alterations created a new biological function for the beta domain, which lacked activating activity in the cellular assays. It has also been reported that α-lactalbumin forms high molecular weight complexes with oleic acid [43]. Such complexes, which also include complex formed from equine lysozyme [50], contain much higher amounts of oleic acid than HAMLET and may thus perturb cell membranes in a manner that resembles lysis rather than programmed cell death.

HAMLET is rapidly internalized by tumor cells, which change morphology, with rounding up, loss of cytoplasm and nuclear condensation followed by death. The alpha-domain peptide-oleate mixtures reproduced these end points. They were rapidly internalized and caused significant changes in cell morphology and loss of viability. Several of these responses may be attributable to ion channel activation as ion channel inhibitors block HAMLET internalization, morphologic change, gene expression and death (unpublished). Like HAMLET, the alpha domain peptide-oleate mixtures activated Na^+^, K^+^ and Ca^2+^ fluxes and while these responses were weaker than in HAMLET treated cells, they were qualitatively and quantitatively different from the responses to sodium oleate alone. Sodium oleate alone only activated K^+^ fluxes, suggesting that the peptides extend the ion channel repertoire, despite being inactive on their own. Weaker cellular responses might reflect a partial attack of each peptide compared to HAMLET. Preliminary results suggest that when the two alpha domains were combined, ion channel activation was comparable to HAMLET, suggesting that several alpha-HAMLET epitopes may act in concert and that multiple interactions may facilitate cellular attack.

Independent, cell-penetrating properties were detected in peptides 1 and 11, from the alpha1 and alpha2 domains, respectively (Fig. S6). These peptides triggered K^+^ fluxes, suggest direct effects on tumor cell membranes. Peptide 11 also triggered Ca^2+^ fluxes, suggesting a difference in specificity between peptides 1 and 11. It is interesting to speculate that different peptides activate different facets of the HAMLET ion flux repertoire. The Na^+^, K^+^ and Ca^2+^ fluxes accompanying cell death were reproduced by the alpha1- and alpha2 peptides, which were cytotoxic in the presence of sodium oleate. The smaller peptides triggered a more restricted repertoire of ion fluxes and were not cytotoxic.

HAMLET is the first example of a protein that in its native state exhibits a well-defined function but also acquires a different, beneficial function after partial unfolding [11], [51]. The properties of HAMLET suggest a mechanism of structure-function variation, which might generate significant structural diversity to meet functional demands in different tissues. Moonlighting proteins, in contrast, serve one main and several additional functions, while undergoing a structural change to a different, well-folded state [52]. More recently, intrinsically unstructured proteins were included in the moonlighting group [53]. These proteins lack a well-defined tertiary structure but can fulfill specific biological functions. HAMLET is distinct from the moonlighting proteins and from the intrinsically unfolded proteins, because it undergoes partial unfolding and retains a defined three dimensional shape to fulfill its new biological function as shown by the determined low resolution solution structure of HAMLET. The low resolution SAXS structure presented here supports the notion of HAMLET as a largely monomeric molecular entity with disrupted globularity, alteration of its tertiary structure in the domain remaining globular and gain of a tail domain due to an extension of the C-terminal portion of the molecule.

## Supporting Information

Figure S1 **Determination of the native molecular mass by gel filtration analysis.** (A), Superdex 75 gel filtration analysis of HAMLET was performed as described under “Materials and Methods”. The insert shows an SDS-PAGE of the HAMLET fractions (grey area in the chromatogram), which have been used for the SAXS experiments. (B) Proteins used as molecular size standards (⧫) were BSA ((*I*), 67 kDa), ovalbumin ((*II*), 45 kDa), β-chymotrypsin A ((*III*), 25 kDa), ribonuclease A ((*IV*), 13.7 kDa) and subunit F (15 kDa) from the *Methanosarcina mazei* Gö1 A-ATP synthase (**o**). (*B*), for each protein, a *Kav* parameter was derived as described under “EXPERIMENTAL PROCEDURES”. The *Kav* for HAMLET is indicated by (**▪**).
(TIF)

Figure S2 **Transmission light DIC images of cells in** **Fig. 4A** **.** A549 cells incubated for 1 h with alpha1-oleate or alpha2-oleate show a round morphology, while cells incubated with oleate-free alpha1-, alpha2-, beta peptides or with oleate remain as flat and extended.
(TIF)

Figure S3 **Quantification of peptide internalization.** Low magnification images of A549 cells incubated with DMSO, alpha1-, alpha2- or beta peptides in the presence (A) or absence (B) of oleate. The alpha1 and alpha2 peptides are internalized, if incubated together with oleate. Without oleate, the peptides are not internalized. Scale bar 100 µm. (C) Quantification of internalization as measured by fluorescence intensity.
(TIF)

Figure S4 **Quantification of red fluorescence intensity for cells in** **Figure 6A** **by fluorescence microscopy.**
(TIF)

Figure S5 **Effects of peptides on the viability of A549 lung carcinoma cells.** Cell viability was quantified as ATP levels (A) and PrestoBlue (B) in % of control after 3 hours of incubation.
(TIF)

Figure S6 **Mapping of biologically active extended peptides.** Peptide 1 (red) and peptide 11 (blue) are highlighted in the crystallographic structure of human α-lactalbumin (PDB id: 1B9O [21]).
(TIF)

Table S1 **A summary of the biological activities of the extended peptides.**
(DOC)
