# Supplementary figures and images for: Low Resolution Solution Structure of HAMLET and the Importance of Its Alpha-Domains in Tumoricidal Activity
Source: PLoS One. 2012 Dec 27;7(12):e53051. doi: 10.1371/journal.pone.0053051 (PMC3531425; doi:10.1371/journal.pone.0053051)

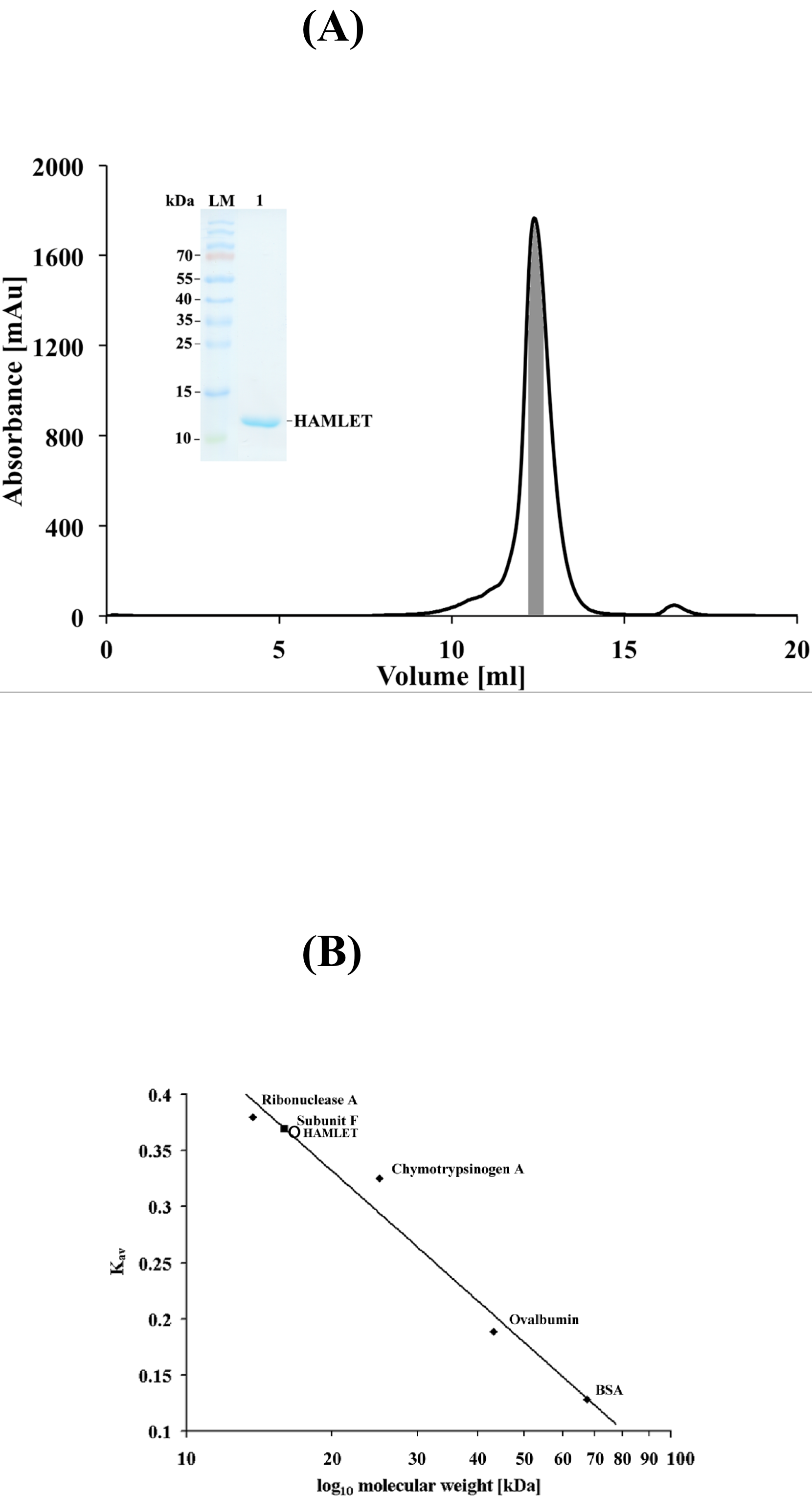

Supplement: Figure S1 — Determination of the native molecular mass by gel filtration analysis. (A), Superdex 75 gel filtration analysis of HAMLET was performed as described under “Materials and Methods”. The insert shows an SDS-PAGE of the HAMLET fractions (grey area in the chromatogram), which have been used for the SAXS experiments. (B) Proteins used as molecular size standards (⧫) were BSA ((I), 67 kDa), ovalbumin ((II), 45 kDa), β-chymotrypsin A ((III), 25 kDa), ribonuclease A ((IV), 13.7 kDa) and subunit F (15 kDa) from the Methanosarcina mazei Gö1 A-ATP synthase (o). (B), for each protein, a Kav parameter was derived as described under “EXPERIMENTAL PROCEDURES”. The Kav for HAMLET is indicated by (▪). (TIF) [file pone.0053051.s001.tif]

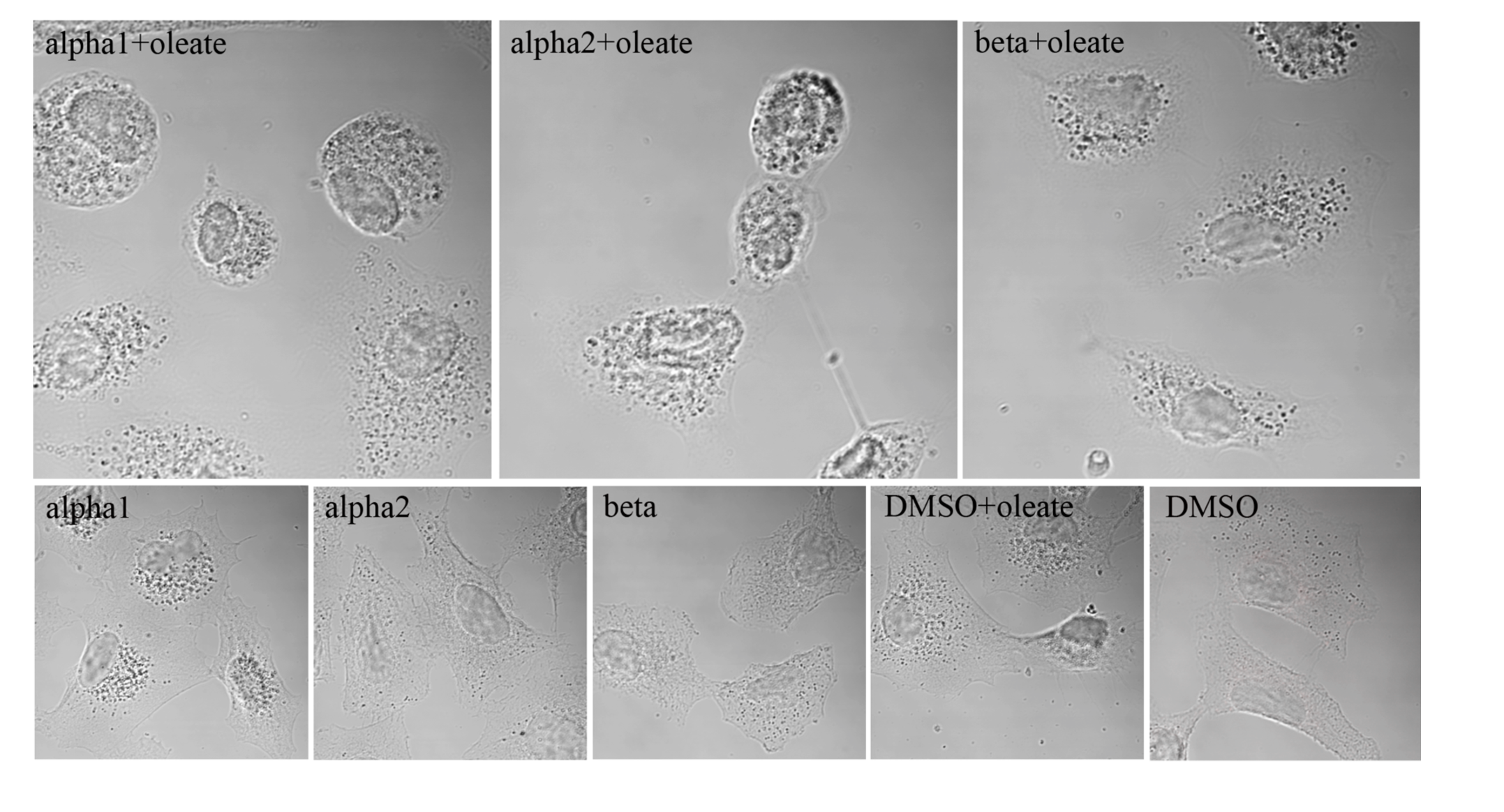

Supplement: Figure S2 — Transmission light DIC images of cells in Fig. 4A . A549 cells incubated for 1 h with alpha1-oleate or alpha2-oleate show a round morphology, while cells incubated with oleate-free alpha1-, alpha2-, beta peptides or with oleate remain as flat and extended. (TIF) [file pone.0053051.s002.tif]

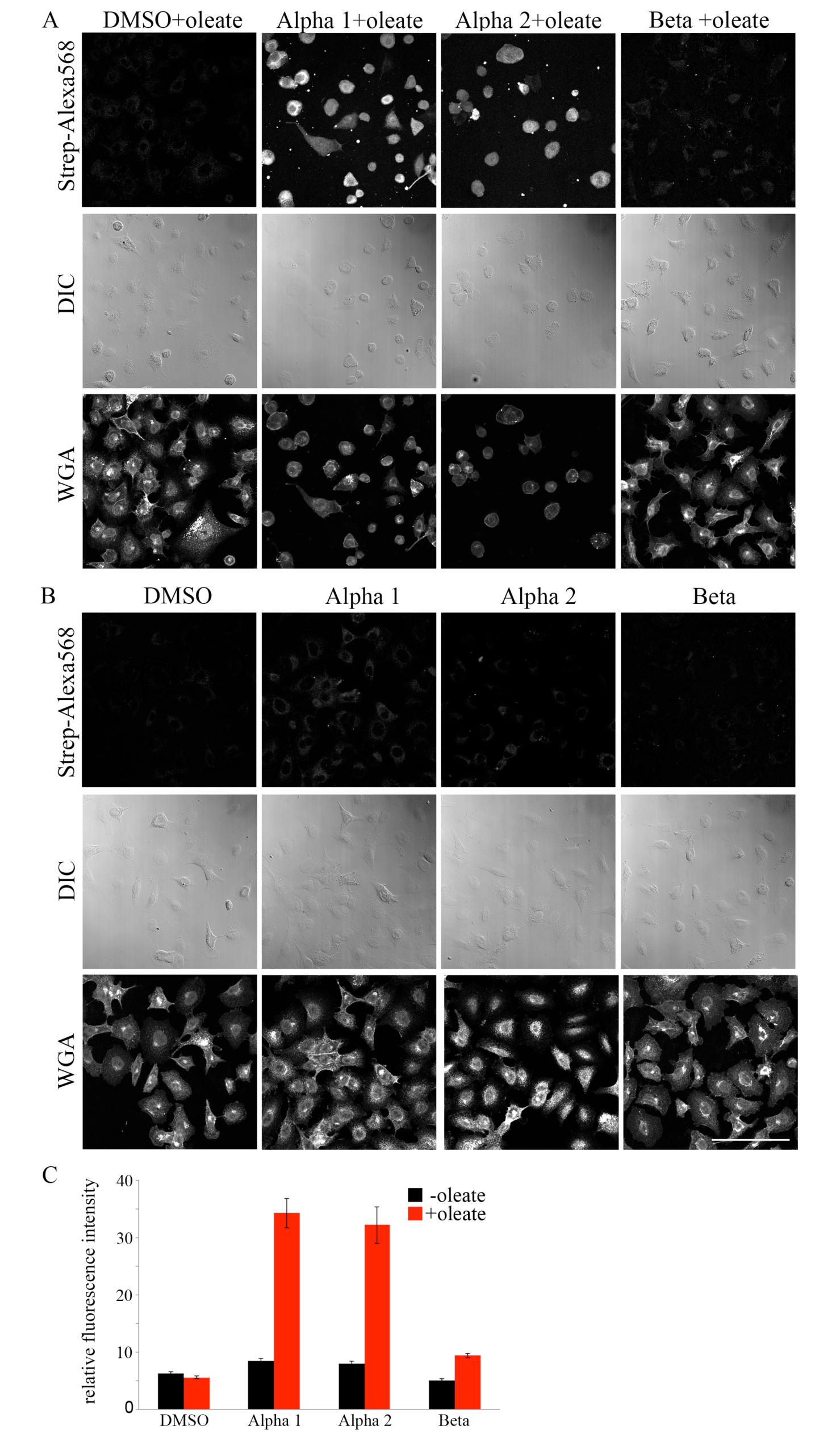

Supplement: Figure S3 — Quantification of peptide internalization. Low magnification images of A549 cells incubated with DMSO, alpha1-, alpha2- or beta peptides in the presence (A) or absence (B) of oleate. The alpha1 and alpha2 peptides are internalized, if incubated together with oleate. Without oleate, the peptides are not internalized. Scale bar 100 µm. (C) Quantification of internalization as measured by fluorescence intensity. (TIF) [file pone.0053051.s003.tif]

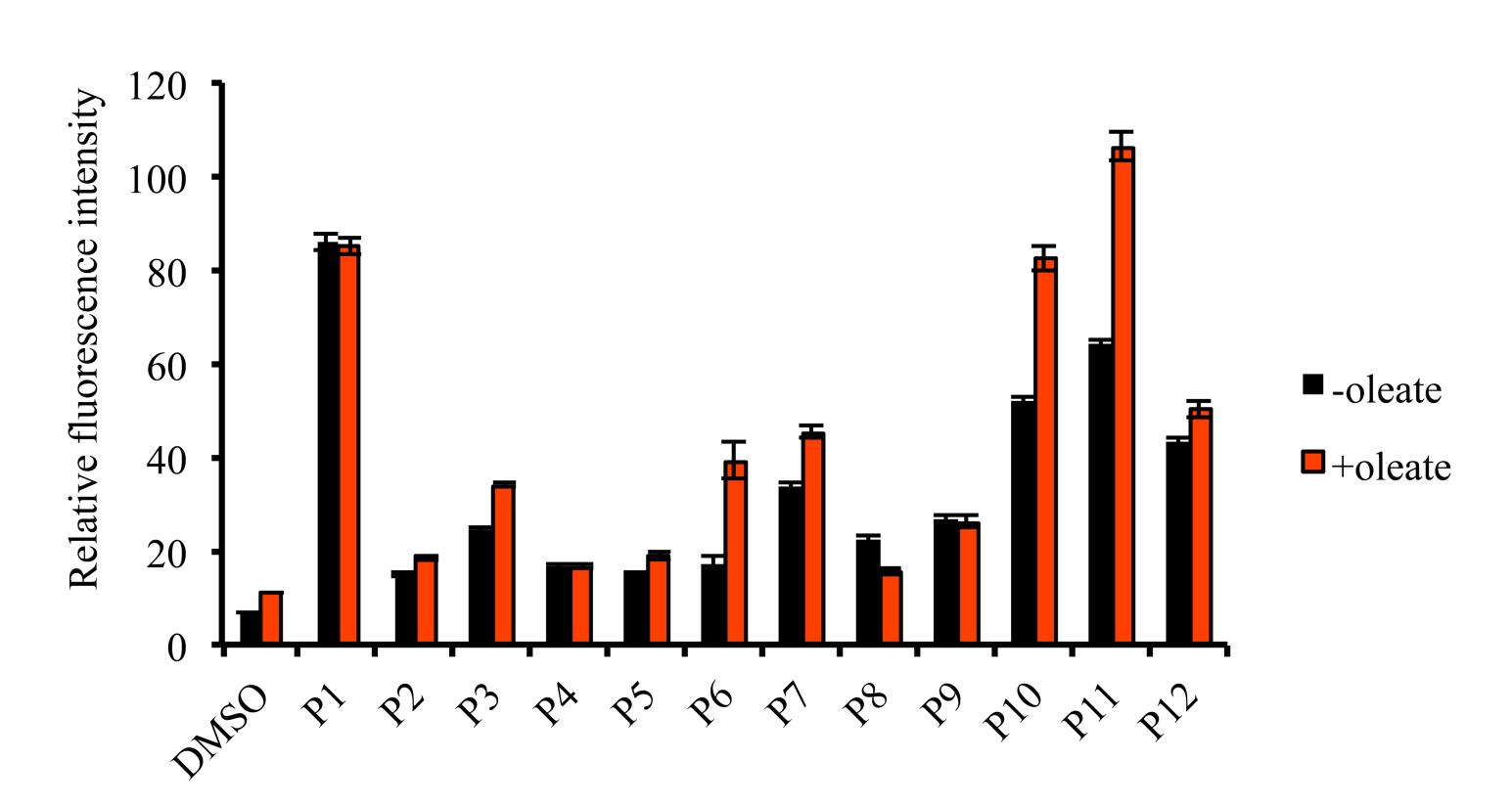

Supplement: Figure S4 — Quantification of red fluorescence intensity for cells in Figure 6A by fluorescence microscopy. (TIF) [file pone.0053051.s004.tif]

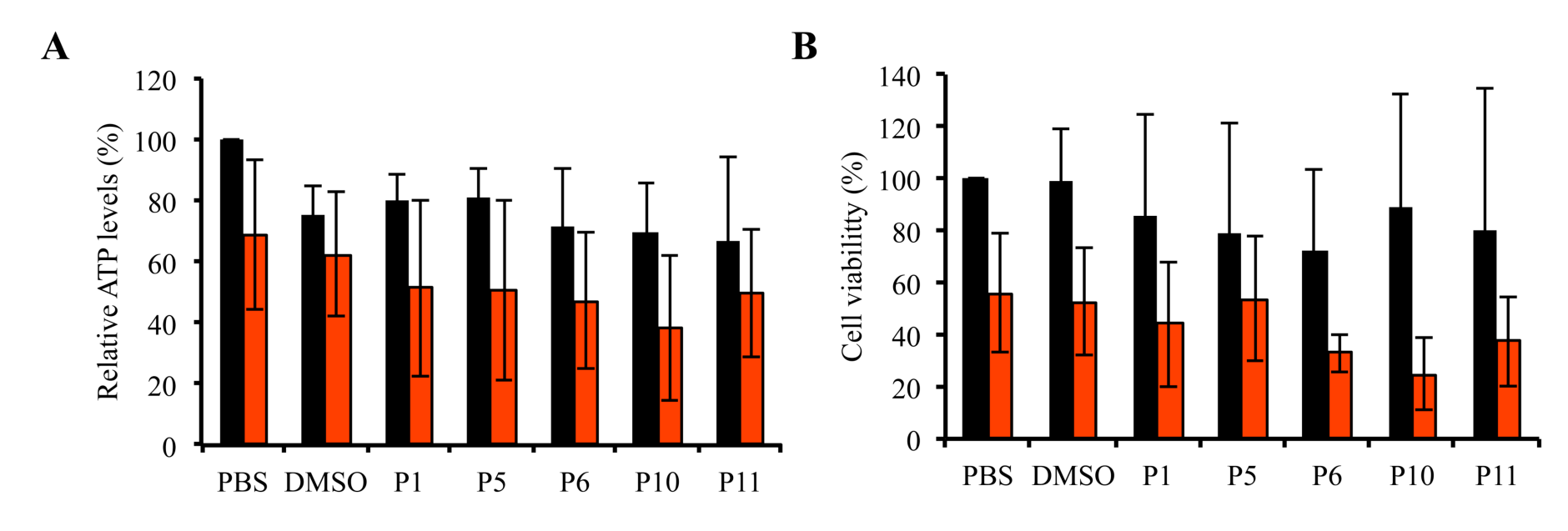

Supplement: Figure S5 — Effects of peptides on the viability of A549 lung carcinoma cells. Cell viability was quantified as ATP levels (A) and PrestoBlue (B) in % of control after 3 hours of incubation. (TIF) [file pone.0053051.s005.tif]

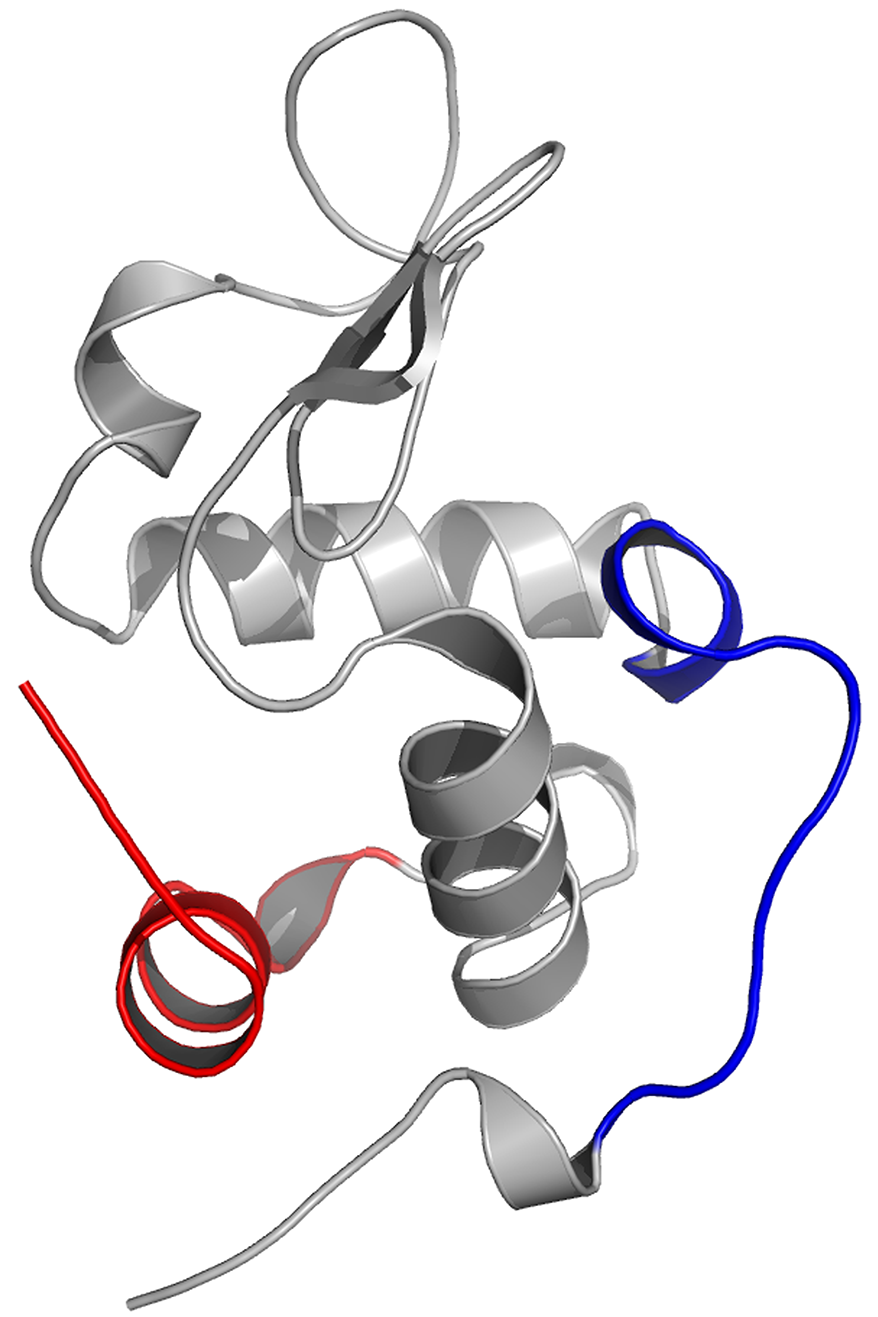

Supplement: Figure S6 — Mapping of biologically active extended peptides. Peptide 1 (red) and peptide 11 (blue) are highlighted in the crystallographic structure of human α-lactalbumin (PDB id: 1B9O [21]). (TIF) [file pone.0053051.s006.tif]
