# Supplementary material for: Low Resolution Solution Structure of HAMLET and the Importance of Its Alpha-Domains in Tumoricidal Activity
Source: PLoS One. 2012 Dec 27;7(12):e53051. doi: 10.1371/journal.pone.0053051 (PMC3531425; doi:10.1371/journal.pone.0053051)
Supplement: Table S1 — A summary of the biological activities of the extended peptides. (DOC) [file pone.0053051.s007.doc]

Supplementary table 1. A summary of biological effect of the extended peptides

| **Peptide** | **Tumor cell uptake** | | **K^+^ flux** | | **Ca^2+^ flux** | | **Na^+^ flux** | |
| --- | --- | --- | --- | --- | --- | --- | --- | --- |
|  | - oleate | + oleate | - oleate | + oleate | - oleate | + oleate | - oleate | + oleate |
| **Peptide 1** | Yes | Yes | Yes | Yes | No | No | No | No |
| **Peptide 2** | No | No | No | No | No | No | No | No |
| **Peptide 3** | No | No | No | No | No | No | No | No |
| **Peptide 4** | No | No | No | No | No | No | No | No |
| **Peptide 5** | No | No | No | No | No | No | No | No |
| **Peptide 6** | No | No | No | No | No | Weak | Weak | Weak |
| **Peptide 7** | No | No | No | No | No | No | No | No |
| **Peptide 8** | No | No | No | No | No | No | No | No |
| **Peptide 9** | No | No | No | No | No | No | No | No |
| **Peptide 10** | Yes | Yes | No | No | Weak | Weak | Weak | Weak |
| **Peptide 11** | Yes | Yes | Yes | Yes | Yes | Yes | No | No |
| **Peptide 12** | No | No | No | No | No | No | No | No |
